# Adipsic Diabetes Insipidus—The Challenging Combination of Polyuria and Adipsia: A Case Report and Review of Literature

**DOI:** 10.3389/fendo.2019.00630

**Published:** 2019-09-18

**Authors:** Rinkoo Dalan, Hanxin Chin, Jeremy Hoe, Abel Chen, Huiling Tan, Bernhard Otto Boehm, Karen SuiGeok Chua

**Affiliations:** ^1^Department of Endocrinology, Tan Tock Seng Hospital, Singapore, Singapore; ^2^Metabolic Medicine, Lee Kong Chian School of Medicine, Nanyang Technological University Singapore, Singapore, Singapore; ^3^Department of Medicine, Yong Loo School of Medicine, National University of Singapore, Singapore, Singapore; ^4^Department of Anaesthesiology, Intensive Care and Pain Medicine, Tan Tock Seng Hospital, Singapore, Singapore; ^5^Department of Rehabilitation Medicine, Tan Tock Seng Hospital, Singapore, Singapore

**Keywords:** adipsia, diabetes insipidus, water balance, thirst and drinking, hypothalamus and neuroendocrinology

## Abstract

Adipsic Diabetes Insipidus is a rare hypothalamic disorder characterized by a loss of thirst in response to hypernatraemia accompanied by diabetes insipidus. These occur secondary to a congregation of defects in the homeostatic mechanisms of water balance. A 27-year old Chinese female presented with Adipsic Diabetes Insipidus after cerebral arteriovenous malformation (AVM) surgery. Initial diagnosis and management was extremely challenging. Long term management required a careful interplay between low dose vasopressin analog treatment and fluids. Detailed charts of medication and sodium balance are described in the case presentation. We performed a literature search of similarly reported cases and describe the possible pathogenesis, etiology, clinical presentation, acute and chronic management, and prognosis.

## Background and Introduction

Hypothalamic Adipsic Diabetes Insipidus (ADI), a rare syndrome with heterogenous clinical presentation, is characterized by a congregation of defects in the homeostatic mechanisms for water balance. These include osmoregulation of thirst mechanism, arginine vasopressin secretion, and renal ability to concentrate urine. Less than one hundred severe cases have been reported worldwide over the last four decades (1).

The management of such patients is extremely challenging as these patients tend to suffer from wide swings in plasma sodium even when the DI is well-controlled (2).

We report a case of severe ADI which occurred postoperatively after cerebral arteriovenous malformation (AVM) surgery. We describe the possible pathogenesis in this case and review the literature for all similarly reported cases and describe the potential mechanisms involved, clinical presentation, acute as well as chronic management, and prognosis.

## Case Presentation

A 27-year-old lady presented with left frontal intracerebral hemorrhage with intraventricular extension secondary to a basal ganglia large complex (AVM) with complex arterial feeders from left middle cerebral arteries (MCA), and anterior cerebral arteries (ACA) with Spetzler Martin Grading Scale of 3 (3).

After initial recovery from decompressive craniotomy and clot evacuation surgery, she underwent an elective AVM excision surgery ~2 weeks later. During the AVM surgery, the surgeons reported brisk bleeding from the feeder vessels (the MCA, ACA, and the choroidal vessels) with severe intracerebral hemorrhage and intra-ventricular hemorrhage requiring massive blood transfusion of up to 32 units intraoperatively. She was placed on barbiturate-induced coma and induced hypotension to minimize cerebral oedema (4).

She started to have polyuria with large volumes of urine intra-operatively soon after the episode of profuse intraoperative bleeding. Although pre-operatively her sodium concentrations were normal, sodium concentrations were higher immediately (within the 1st hour) postoperatively within the range of 151–160 mmol/L in tandem with a low urine osmolality <130 mosm/L. Transient DI was considered and she was started on intravenous desmopressin (IV desmopressin) 1 mcg which was given daily along with 3.5–4 L half strength saline infusion for 3 consecutive days.

However, she since developed pulmonary oedema (likely a consequence of massive blood transfusion intraoperatively, intravenous hypotonic fluids, and concurrent IV desmopressin) on postoperative day 4, IV desmopressin was discontinued, and fresh frozen plasma and diuretics (intravenous furosemide 40 mg) were administered to reduce the extra-vascular oedema. In the subsequent hour, she started to develop polyuria again with a urine output of 800 mL in a single hour and her sodium concentrations up trended to 172 mmol/L. In view of the concomitant postoperative cerebral oedema, it was decided to lower the sodium concentrations in a targeted manner such that rate of decrease in sodium remained within 5–10 mmol/L/day, with a target level of 155 mmol/L. A low dose of IV desmopressin 0.25 mcg was administered, and titration regime suggested with a view to maintain an hourly urine output of lower than 100 mL/h. She required IV 0.75 mcg desmopressin on postoperative day 5, and then IV 0.25 mcg twice daily on day 6 when she reached a target level of 155 mmol/L. This sodium concentration could be maintained with the desmopressin dosing for another 2 days.

On Day 9, postoperatively, desmopressin was discontinued as she was able to maintain a low urine output without desmopressin, and sodium concentrations were stable. At this stage, it was deemed that she had developed syndrome of inappropriate anti diuretic hormone secretion (SIADH) and fluids were restricted to 1 L/day. Her sodium concentrations remained stable at 134–138 mmol/L during this week.

**Figure 1:**
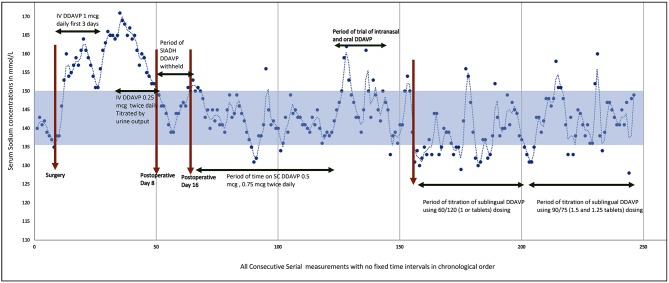
Graph showing all serial measurements in our patient throughout the hospitalization.

On postoperative Day 14, she developed polyuria again with rising sodium concentrations and serum osmolality. At this time IV desmopressin was resumed at 0.25 mcg twice daily. The sodium concentrations could be maintained at 138–150 mmol/L with IV desmopressin (0.25 mcg twice daily) and fluids. She had gone through a triphasic phase with a period of transient DI, followed by SIADH, and subsequent permanent DI (see Figure 1). The serum cortisol, growth hormone, insulin like growth factor-1, thyroid function, and prolactin levels were all within the normal range.

After a ventriculoperitoneal shunt insertion (done to maintain intracranial pressure) 6 weeks after the AVM excision, she was transferred to rehabilitation wards and the intravenous desmopressin regime converted to a subcutaneous (SC) regime (see Figure 1). When nasogastric tube feeding was discontinued, we tried to regulate fluids by thirst. Blood investigations showed rising sodium levels up to 165 mmol/L and she felt no thirst during this period of hypernatraemia despite a serum osmolality of 350 mosm/kg. She remained adipsic despite recovery of aphasia. A thirst scale was not used as upon questioning, she verbalized that she felt no thirst at all, and a thirst scale could not be applied. Hence, fluids had to be regulated to maintain sodium balance. Her urine output was fixed and her sodium balance well-regulated on a SC regime of desmopressin 0.5 mcg in the morning and 0.375 mcg at night, with regulated fluids of 1.5–2 L per day. She did not have any evidence of thermoregulatory disturbances. Polyetheretherketone (PEEK) cranioplasty was performed ~4 months after initial surgery.

After 5 months of intensive multidisciplinary rehabilitation, she still had a non-functional dominant right hand and significant residual cognitive deficits, and safe dosing and self-administration of subcutaneous desmopressin was not possible in the long term.

A trial of nasal desmopressin and oral desmopressin showed significant variable absorption leading to greater fluctuations in the sodium concentrations. Hereafter, we decided to use the sublingual desmopressin available as desmopressin Melt. After several trials of different dosing regimens we were able to fix the urine output to approximately 2 L/day using desmopressin Melt oral lyophilizate 120 mcg (2 tablets) in the morning and 60 mcg (1 tablet) in the evening. However, at this dose she was having hyponatremia at certain times of the day. We performed further dose titration by cutting the desmopressin tablets using tweezers/forceps to lift the tablets from the blister pack and cut the tablets into exact quarter pieces (each 15 mcg) with the help of a pill cutter. With a regime of 90 mcg (1.5 tablets) in the morning and 75 mcg (1.25 tablets) in the evening we managed to achieve similar results as with the subcutaneous regime. With this regime, and a regulated fluid intake of 2 L/day, her sodium concentrations were maintained within the range of 135–150 mmol/L without major fluctuations or cognitive symptoms. This dosing also allowed her to have increased activity in the day time for rehabilitation and she was able to sleep well-undisturbed by polyuria at night.

See Figure 1 for the serial sodium measurements in this patient throughout the hospitalization period with the above events described.

In view of the clinical history and the fact that her pituitary and hypothalamus is mostly intact, it is unlikely that she had significant defects in baroregulation.

## Discussion

### Pathophysiology of ADI

In physiological conditions, plasma sodium, osmolality, and water balance are maintained within narrow ranges secondary to a careful interplay between thirst and water intake, neurohypophyseal vasopressin secretion, and the antidiuresis at the renal distal collecting-tubules.

Peripheral signals (oropharyngeal, gut osmosensors, blood osmolality) encode information about the current state of hydration in real time and these are centrally integrated (5). A state of dehydration will lead to stimulatory signals to the subfornical-organ (SFO) which hierarchically sends signals to the organum-vasculosum of lamina terminalis (OVLT), and the median preoptic nucleus (MnPO) (6). The MnPO which may serve as a “central detector” sends stimulatory signals to the hypothalamic nuclei to increase release of vasopressin and also sends increased thirst signal through the SFO and OVLT (7, 8). The thirst related neural pathways connecting the SFO and OVLT to the cingulate and insular cortex results in the conscious perception of thirst (7, 8). On the contrary in the state of adequate hydration, the signals from the peripheral sensors, leads to an inhibition of these areas, and negative feedback from MnPO leading to suppression of thirst and lower vasopressin secretion and release (red circuit). In effect, neurons in the MnPO, OVLT, and SFO are extensively and reciprocally interconnected since optogenetic and chemogenetic activation of neurons in any of these areas can stimulate thirst (9). Hence, disruption of blood supply resulting in cell damage will lead to adipsia. Cases of ADI due to autoimmunity to neuronal cells from the SFO and OVLT have also been published indicating the functional importance of these neuronal sensors in thirst control (10).

The AVP neurons exhibit “phasic” activity of action potentials with intervals wherein AVP is released at the axon terminals. The release of vasopressin is regulated by the osmosensory inputs from the MnPO, through baroreceptors and there is also an anticipatory regulation wherein vasopressin release is stimulated before meals (prandial), on hyperthermia and in a circadian pattern (11). There is a circadian daily pattern of secretion of vasopressin in the circulation with an increase in levels between 24:00 and 02:00 h and levels fall progressively during the day with the nadir between 16:00 and 20:00 h (12). This circadian midnight increase caused by an increase in vasopressin release results in part from enhanced synaptic excitation of magnocellular neurosecretory neurons by the suprachiasmatic nucleus which is known to regulate circadian rhythm (11). In ADI the osmosensory regulation of vasopressin secretion is most affected and other effects maybe variable depending on the site and extent of injury whereas baroreceptor response is known to be preserved in many patients with limited injury as demonstrated in one study (13).

AVP acts on renal collecting ducts via Vasopressin-2 (V2) receptors to increase water permeability which leads to decreased urine formation. The reabsorption of water is controlled through regulation of the water channel, aquaporin-2 (AQP2). AVP induces in the short-term, intracellular translocation of AQP2-bearing vesicles to the apical plasma membrane thus regulating AQP2 trafficking. In the long term with adequate AVP concentrations, it regulates the transcription of the AQP2 gene to increase AQP2 protein abundance in the collecting duct (14). In ADI, we postulate that it is likely that the acute osmosensory response is affected more than the chronic response. Hence there will be a relative abundance of aquaporin but the acute trafficking to the apical membrane will be affected.

**Figure 2:**
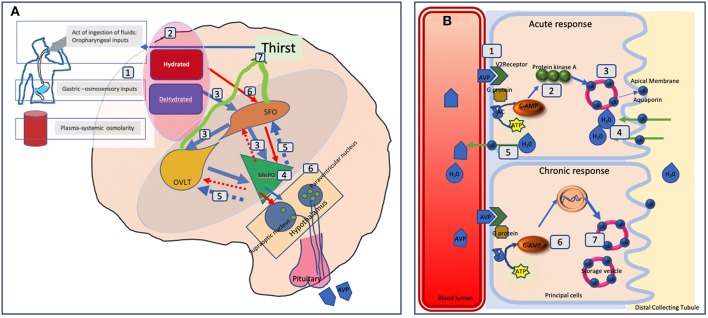
Schematic representation of fluid balance regulation in humans. **(A)** The control and regulation of vasopressin secretion in the brain. **(A)** Peripheral signals (oropharyngeal, gut osmosensors, blood osmolality) encode information about the current state of hydration in real time (1) and centrally integrated (2). A state of dehydration leads to stimulatory signals to the SFO which hierarchically sends signals to the OVLT and the MnPO (3). The MnPO which may serve as a “central detector” sends stimulatory signals to the hypothalamic nuclei to increase release of vasopressin (4), and also sends increased thirst signal through the SFO, and OVLT (5, blue circuit in the diagram). On the contrary in the state of adequate hydration signals from the peripheral sensors, there is an inhibition of these areas and negative feedback from MnPO leading to suppression of thirst and lower vasopressin secretion and release (red circuit 6). The thirst related neural pathways connecting the SFO and OVLT to the cingulate and insular cortex results in the conscious perception of thirst (7). In adipsic DI, in the absence of osmosensory stimuli associated neural circuits due to cell damage, stimulatory release, or inhibition of vasopressin and the conscious perception of thirst both are disrupted. **(B)** The mechanism of action of vasopressin on the renal collecting tubules. **(B)** Vasopressin in the blood circulation attaches to Vasopressin receptor 2 on the luminal surface of the principal cells of the distal collecting tubule in the kidneys (1). In the acute phase, via synthesis of cAMP from ATP through AC (adenylate cyclase) it forms protein kinase A (2) which stimulates the translocation of aquaporin from the storage vesicle to the apical membrane and blood luminal surface (3). This results in water being transported into the cell (4) and absorbed subsequently into the circulation (5). In the chronic phase, via cAMP mediated transcriptional control of the aquaporin gene (6) increased levels of the aquaporin water channel can be found in the principal cells (7). In adipsic DI, the acute response is likely to be more affected than the chronic response as the osmosensory related vasopressin secretion is affected.

See Figure 2 for detailed illustration of the mechanisms.

### Literature Search

We performed a systematic literature search including Pubmed, Scopus, and Embase for cases reported as a case report or case series of ADI from years 1980 to 2018. We used the search term: *Adipsia, Adipsic disorder, and Adipsic DI* and we limited the search to English language and to Humans. We also searched any other references identified during reading of each case report/case series. We found 25 references (10, 13, 15–39) with a total of 45 cases reported in the literature. We have summarized all the case reports in Table 1.

**Table 1:** Demographics, etiology, clinical presentation, management, and prognosis of patients with adipsic diabetes insipidus reported in the literature.

**No**	**References**	**Age (years)/Gender**	**Etiology**	**Treatment**	**Clinical presentation**	**DDAVP dose**	**Anterior pituitary defects**	**Prognosis (Recovery of DI)**
1	Cuesta et al. (15)	51/Male	ACOM aneurysm	Surgical Repair of aneurysm	Acute polyuria after surgery	Oral DDAVP dose 200 mcg thrice daily	Hypogonadotrophic hypogonadism and Secondary Hypothyroidism	Recovered after 10 years
2	Imai et al. (16)	38/Male	ACOM aneurysm	Surgical Repair of aneurysm	Acute polyuria after surgery	Intranasal DDAVP 30 mcg/day	Not known	Remission after 1 year
3	McIver et al. (17)	39/Female	ACOM aneurysm	Surgical Repair of aneurysm	Acute polyuria after surgery	Intranasal DDAVP twice daily	Not reported	No
4	McIver et al. (17)	30/Male	ACOM aneurysm	Surgical Repair of aneurysm	Acute polyuria after surgery	Intranasal DDAVP twice daily	Not reported	Not reported
5	Ball et al. (18)	28/Male	ACOM aneurysm	Surgical Repair of aneurysm	Chronic presentations with abnormal sodium and loss of thirst.	Only required fluids with no DDAVP	Normal	Not reported
6	Nguyen et al. (19)	46/Male	ACOM Aneurysm	Surgical Repair of aneurysm	10 days after operation sodium found to be high at 167 mmol/L	Intranasal DDAVP 20 mcg twice daily, chlorpropamide 250 mg and Hydrochloro-thiazide	Normal	Not Reported
7	Smith et al. (13)	39/Female	ACOM aneurysm	Surgical Repair of aneurysm	Acute polyuria after surgery	Not reported	None	Not reported
8	Smith et al. (13)	30/Male	ACOM aneurysm	Surgical Repair of aneurysm	Acute polyuria after surgery	Not reported	None	Not reported
9	Smith et al. (13)	28/Male	ACOM aneurysm	Surgical Repair of aneurysm	Acute polyuria after surgery	Not reported	None	Not reported
10	Smith et al. (13)	40/Male	ACOM aneurysm	Surgical Repair of aneurysm	Acute polyuria after surgery	Not reported	None	Not reported
11	Nussey et al. (20)	30/ Male	ACOM aneurysm	Surgical Repair of aneurysm	Acute polyuria after surgery	Not reported	Thermoregulatory disturbances and hypothermia	Not reported
12	Spiro and Jenkins (21)	52/Female	ACOM aneurysm	Surgical Repair of aneurysm	Acute polyuria after surgery	Not reported	Thermoregulatory disturbances and hypothermia	None
13	Crowley et al. (22)	Case Series of 13 patients: 8/13 Males Age: 16–56 years	ACOM aneurysm (n-4), craniopharyngioma (n-4), brain trauma (n-1), Toluene (n-1), neurosarcoidosis (n-1), pituitary tumor (n-1), and congential (n-1)	Depending on pathology	Acute polyuria after surgery for postoperative and trauma cases.	Not reported	8 patients had Panhypopituitarism	No remission Demise due to respiratory failure (3/13 died)
14	Cuesta et al. (15)	41/Female	Craniophayrngioma and Left Para arachnoid Aneurysm	Surgical Repair of aneurysm	Acute polyuria after surgery	DDAVP dose 200 mcg twice daily	Panhypopituitarism	Adipsia recovered in 2 years but DI persisted
15	Cuesta et al. (15)	29/Female	Craniopharyngioma	Surgical excision with complicated subdural hematoma and cerebral shunt insertion	Acute polyuria after surgery	Oral DDAVP 200 mcg thrice daily	Panhypopituitarism requiring cortisol,	Progressed to compulsive water drinking after 8 years
16	Colleran et al. (23)	18/Male	Craniopharyngioma	Surgical treatment	Presentation is 5 years after initial surgery	IV DDAVP 1 mcg twice daily	Panhypopituitarism	No, obesity
17	Zantut-Wittmann et al. (24)	34/Male	craniopharyngioma	Transfrontal resection	Acute polyuria after surgery	DDAVP 30 mcg IN along with regular fluids more than 2 L /day	Normal	Not reported
18	Raghunathan et al. (25)	11/Male	Craniopharyngioma	Transfrontal excision	Acute polyuria after surgery	DDAVP 10 μg puff IN every 24–36 h upon breakthrough with 1.2–1.5 L fluids	Panhypopituitarism	Not Reported
19	Smith et al. (13)	18/Female	Craniopharyngioma	Surgery	Acute polyuria after surgery	Not reported	Panhypopituitarism	Not reported
20	Smith et al. (13)	16/Female	Craniopharyngioma	Surgery	Acute polyuria after surgery	Not reported	Panhypopituitarism	Not reported
21	Smith et al. (13)	56/Male	Craniopharyngioma	Surgery	Acute polyuria after surgery	Not reported	Panhypopituitarism	Not reported
22	Sinha et al. (26)	15/Male	Craniopharyngioma	Surgical Resection	Acute polyuria after surgery	Not reported	Panhypopituitarism	Remission in 7 months
23	Sinha et al. (26)	13/Male	Craniopharyngioma	Surgical Resection	Acute polyuria after surgery	Not reported	Panhypopituitarism	Remission d in 9 months
24	Sinha et al. (26)	11/Male	Craniopharyngioma	Surgical Resection	Acute polyuria after surgery	Not reported	Panhypopituitarism	Remission in 4 months
25	Sinha et al. (26)	11/Male	Cavernous haemangioma with intracranial hemorrhage	Stereotactic radiotherapy to the angioma	Polyuria with loss of thirst	DDAVP 100 mcg twice daily orally	Radiation induced encephalopathy and visual failure	Not reported
26	Pérez et al. (27)	24/Female	Craniopharyngioma	Transcranial surgery	Polyuria after surgery	DDAVP melts 60 mcg om and 120 mcg on	Panhypopituitarism	Not Reported
27	Pabich et al. (28)	16/Female	Craniopharyngioma	Transsphenoidal surgery	Polyuria and adipsia after surgery	SC DDAVP 1 mcg twice daily	Panhypopituitarism	Not reported
28	Pereira et al. (29)	13/Female	Germinal Cell tumor of the hypothalamus	Radiotherapy	6 months after RT	DDAVP sublingual, 0.72 mcg/day	Panhypopituitarism	no
29	Sinha et al. (26)	15/Male	Pineal tumor extending into the third ventricle and infiltrating along the walls of the lateral ventricle	Radiotherapy to the entire neuroaxis	First presented with adipsic polyuria	Fixed dose of DDAVP and sliding scale fluids	Panhypopituitarism	Not reported
30	Zhang et al. (30)	16/Male	Hypothalamic hamartoma.	Surgery	Chronic -presented 10 years after surgery	DDAVP 0.05 mg (50 mcg) orally every 12 h	Normal	Not reported
31	Latcha et al. (31)	37/Female	H/o hepatocellular Carcinoma with metastasis to hypothalamus. MRI showed enhancing mass centered upon the supra sellar cistern and anterior third ventricle measuring 3.0 × 3.9 × 3.4 cm.	No treatment reported	Chronic presentation	DDAVP oral 50 mcg 2 times a day,	Normal	Not Reported
32	Sherlock et al. (32)	14/Male	Macroprolactinoma	massive intraoperative hemorrhage during three resection surgeries	Acute polyuria after surgery	Oral DDAVP 200 mcg twice daily with 2 L fluids.	Panhypopituitarism	Not reported
33	Ball et al. (18)	14/Female	Null cell pituitary tumor (aggressive) recurrent (2nd time)	Transfrontal adenomectomy	Acute polyuria after surgery	DDAVP 250 mcg twice daily SC	Panhypopituitarism	Demise due to large malignant pituitary tumor
34	Smith et al. (13)	22/Male	Head injury		Acute polyuria	Not reported	None	Not reported
35	Smith et al. (13)	26/Male	Toluene Exposure		Episodes of Adipsic DI	Not reported	None	Not reported
36	Hiyama et al. (10)	6.5/Female	Retroperitonial ganglioneuroma not related as no resolution after resection with no structural abnormality in the hypothalamus/pituitary	Found to autoantibodies to Na_x_ which were also expressed on the tumor cells. These antibodies were seen to react with the circumventricular organs in mice and was postulated to be the cause	Chronic presentation	Controlled Fluids intake only. Did not require DDAVP	None	Not reported
37	Hiyama et al. (33)	9/Female	No structural abnormality in the hypothalamus –pituitary h/o influenza	Found to have antibodies that reacted to mouse SFO (subfornical organ)	Polyuria with absence of thirst	DDAVP and fluids	None	None
38	Hiyama et al. (33)	16/Female	Background opsoclonus myoclonus syndrome No structural lesion of the hypothalamus pituitary region	Found to have antibodies that reacted to mouse SFO (subfornical organ)	Polyuria with absence of thirst	DDAVP and fluids	None	None
39	Hiyama et al. (33)	8/Female	No structural lesion of the hypothalamus pituitary region	Found to have antibodies that reacted to mouse SFO (subfornical organ) and to OVLT	Polyuria with absence of thirst	DDAVP and fluids	None	None
40	Komatsu et al. (34)	16/Male	Developmental delay and mental retardation,	Found to have hypogenesis of corpus callosum	Polyuria with absence of thirst	DDAVP and fluids	Hypothalamic hypogonadism	None
41	Schaff-Blass et al. (35)	8months/Male	Developmental Delay and microcephaly,	Dysplastic Corpus callosum and dysplastic septum pellucidum	Polyuria with absence of thirst	DDAVP and fluids	Hypothalamic hypogonadism and hypothyroidism	None
42	AvRuskin et al. (36)	11/Male	Developmental Delay and microcephaly	Agenesis of Corpus Callosum and dilated ventricles	Polyuria with absence of thirst	DDAVP and fluids	None	None
43	Radetti et al. (37)	4 months Female (2 sisters)	Developmental Delay and microcephaly, West syndrome	Dysplastic Corpus callosum and dysplastic septum pellucidum	Polyuria with absence of thirst	DDAVP and fluids	None	None
44	Takeya et al. (38)	13/Female	Short Stature, Cerebral palsy	Agenesis of Corpus Callosum	Polyuria with absence of thirst	DDAVP and fluids	None	None
45	O'Reilly et al. (39)	22/Male	Neurosarcoidosis	Infliximab	Polyuria and subsequent loss of thirst	DDAVP and fluids	Panhypopituitarism	Complete recovery after Infliximab
46	Our case	27/Female	Arteriovenous malformation	Surgery with postoperative ventricular shunt insertion	Polyuria in the immediate postoperative period	DDAVP melts and fluids	None	None

*ACOM, anterior communicating artery aneurysm; DDAVP, Desmopressin*.

#### Causes of ADI

Forty percent (17/46) of cases of ADI described in the literature occurred secondary to anterior communicating artery (ACOM) aneurysm rupture and post-surgical clipping using a frontal craniectomy approach. In one case the dual pathology of craniopharyngioma and ACOM aneurysm was described (15). The arterial supply of the OVLT has been described to come from four sources (40): (1) a superior median source branching from the anterior communicating artery, (2) and (3) two lateral sources coming from arteries which branch off from each anterior cerebral artery below the anterior communicating artery, and (4) an inferior median source ascending from below the optic chiasm. Characteristically the capillaries lining this organ lies outside the blood brain barrier and hence a change in osmolality can be easily sensed by these organs (41). It should be noted that improvements in surgical techniques and the use of coiling instead of clipping may reduce these complications. For patients in good clinical conditions with ruptured aneurysms of the anterior circulation who are suitable for coiling, data from a meta-analysis show that coiling is associated with a better outcome (42). When compared to clipping wherein both ends of the ACA is clipped with interruption of blood supply to the hypothalamus, the procedure of inserting a coil into the aneurysm is able to preserve the feeders to the hypothalamus in most cases.

The second cause of ADI has been described as suprasellar craniopharyngioma (17/46). These cases often involve a trans-frontal excision and recovery after surgery has been described in some cases. A case of cavernous haemangioma with similar manifestation after bleeding episode has also been described (26).

Hypothalamic tumors inclusive of hamartoma, pineal gland tumor, and germinal cell tumor can present with ADI (26, 29, 30). A case of hepatocellular carcinoma with metastasis to the hypothalamus and resultant ADI has been reported (31).

Interestingly only two cases of pituitary tumors with ADI have been described (18, 32). Both these cases had an aggressive tumor with multiple trans-frontal resection and intracranial hemorrhage intra-operatively. One case each has been described in relation to head injury and toluene exposure as well (13).

Four cases have been described with sudden acute presentation of polyuria and hypernatremia. These patients did not have any structural abnormalities in the hypothalamus and detailed investigations revealed evidence of antibodies against the OVLT organs. These cases have been postulated to have an underlying autoimmune pathology (10, 33).

Congenital or early childhood cases in patients with development problems of corpus callosum have also been described with manifestation of ADI (34–38). A case of neurosarcoidosis extensively affecting the anterior pituitary, posterior pituitary and hypothalamus with ADI has also been described (39).

#### Clinical Presentation of ADI

The clinical presentation in most of the cases described is polyuria in the immediate week after surgical clipping for AVM or after craniotomy. The biochemical presentation is hypernatremia with increased serum osmolality in the presence of inappropriately dilute urine in large volumes. If thirst sensation is assessed using a thirst scale, these ADI patients report low or minimal thirst despite the hypernatremia. Interestingly these patients have a normal response to hypotension and their baroreceptor mechanism is intact (13).

Our patient went through a triphasic phase of initial DI, followed by SIADH and then permanent DI. This pattern described as *triphasic response* has been described after pituitary and craniopharyngioma surgeries. The pathophysiology of the triphasic response appears to be explained by early hypothalamic dysfunction (characterized by polyuria and hypernatremia), subsequent release of preformed vasopressin from the storage vesicles in posterior pituitary (not related to any stimulus) (characterized by SIADH) and, finally, depletion of vasopressin stores (permanent DI) (43). In our patient, it is likely that at the third phase, the deficiency in the response to osmolality in the release of vasopressin is unmasked after SIADH resolves, rather than an absolute depletion of vasopressin.

#### Associated Complications of ADI

Few case reports have described thermoregulatory disturbances. Panhypopituitarism has been reported in craniopharyngioma cases and in pituitary tumor cases only and was not present in our patient.

Although not consistently reported, other important complications of the hypernatremia include deep vein thrombosis; hence low molecular weight heparin may be indicated during acute sickness and immobilization (18).

Hypothalamic obesity and sleep apnoea has been commonly reported as well (18). In one case series (13), wherein they evaluated the AVP response to hypotension, they found that the patients with craniopharyngiomas also had a loss of baro-regulated AVP response. These patients had evidence of panhypopituitarism, indicating that surgery to their tumors had left more widespread damage to the pituitary, including the posterior pituitary which is the final common pathway for AVP secretion.

#### Management Approaches of ADI

The management of these cases has been described (15, 18). Patient and family education on the principles of water balance and management is crucial. Water intake can be fixed to 1.5–2 L daily and then titration of desmopressin performed. Daily weight tracking is useful to detect dehydration or fluid overload. It is recommended to do weekly plasma sodium concentrations to monitor fluctuations. Despite strict monitoring it is difficult to reproduce a round the clock control of plasma sodium concentrations that mimics physiological osmoregulation. In view of risk of rapid swings in sodium concentrations, and the symptoms of hyponatremia seen at the low reference range, it is advisable to keep sodium concentrations within the higher reference range.

Desmopressin the synthetic analog of AVP was first introduced for treatment of DI in 1972 (44). Most patients do well on low doses of desmopressin given twice daily. There is a fine balance and sodium levels can swing to very low levels with minute dose-escalations.

Our patient did very well on very low doses of parenteral administrations which is known to have the lowest variability in bioavailability (45). The absorption of intranasal formulations is highly variable especially in children or adults with cognitive problems as they may not inhale the solution in a consistent manner (46, 47). Oral formulations are a good option as increasing the dose will lead to a longer duration of action but has no peak effect (48–50). We tried this form in varying doses of 50–100 mcg but we found significant day-to day variations in effects with this formulation. Sublingual desmopressin (Minirin Melt) has been available since 2005. Pharmacological studies have demonstrated that sublingual route results in a higher bioavailability by ~60% when compared to oral route (49, 50). Refined doses of sublingual desmopressin could be achieved by cutting each 60 mcg tablet, allowing dose adjustments of 65 to 90 mcg sublingually. Similar pharmacokinetic profile of such split tablets has been described (51). Concomitant intake of food is known to decrease the rate and extent of absorption by 40% hence, it is best to avoid any food consumption within an hour before and after administration of the sublingual desmopressin tablets. Recently detailed fluid administration protocol with daily sodium monitoring with fixed dose subcutaneous desmopressin has been described (28) demonstrating the difficulty in day-to day monitoring. Another recent case report (27) has also reported using the sublingual desmopressin form for ADI at the dose of 60 and 120 mcg daily. The use of the sublingual desmopressin may be a useful alternative in cases where optimum effects cannot be reached with other formulations.These patients require strict monitoring of daily weight, urine volume, and fluid intake round the clock. In view of the cognitive and memory defects, these patients tend to have, they usually need a caregiver to monitor the fluid intake to ensure that they are drinking the required volume.

In our review of cases, 6 patients had a remission of ADI of which 2 were related to ACOM aneurysm (15, 16) and 4 were related to craniopharyngioma (15, 26). One patient with neurosarcoidosis had a remission with Infliximab therapy (39). Such observations may suggest that both osmoreceptors regulating thirst and their efferent pathways demonstrate more neuroplasticity than those regulating AVP production (26).

## Conclusion

ADI is an extremely rare complication of hypothalamic disorders which results in a loss of function in the thirst center. Due to hierarchical control from the thirst center, the absence of thirst is also associated with loss of vasopressin regulation and polyuria. Subjects suffering from ADI need to be delicately managed on low doses of desmopressin and fluid balance. With current monitoring and personalized care approaches, it is still not possible to mimic round- the- clock quasi- physiological osmoregulation.

## Data Availability

All datasets generated for this study are included in the manuscript/supplementary files.

## Ethics Statement

Written informed consent was obtained from the individual(s) for the publication of any potentially identifiable images or data included in this article.

## Consent to Participate

We have obtained written permission from the patient's next of kin to write and publish this manuscript.

## Author Contributions

RD was the primary endocrinologist who managed the patient's fluid balance, conceptualized the idea, wrote the manuscript, did the literature review, acquired the data, wrote, and reviewed the final manuscript. HC, AC, and JH were residents involved in the management of this patient, and reviewed the final manuscript. HT was the anaesthesiologist in charge of this patient during the intensive care phase, conceptualized the idea, and critically evaluated the final manuscript. KC was the principal rehabilitation specialist in charge of the patient she conceptualized the idea, and critically evaluated the final manuscript. BB conceptualized the idea and critically evaluated the final manuscript.

### Conflict of Interest Statement

The authors declare that the research was conducted in the absence of any commercial or financial relationships that could be construed as a potential conflict of interest.
